# Exploring a Novel Multiple-Query Resistive Grid-Based Planning Method Applied to High-DOF Robotic Manipulators

**DOI:** 10.3390/s21093274

**Published:** 2021-05-10

**Authors:** Jesus Huerta-Chua, Gerardo Diaz-Arango, Hector Vazquez-Leal, Javier Flores-Mendez, Mario Moreno-Moreno, Roberto C. Ambrosio-Lazaro, Carlos Hernandez-Mejia

**Affiliations:** 1 Instituto Tecnologico Superior de Poza Rica, Tecnologico Nacional de Mexico, Luis Donaldo Colosio Murrieta S/N, Arroyo del Maiz, Poza Rica, Veracruz 93230, Mexico; chua@itspozarica.edu.mx (J.H.-C.); gerardo.diaz@itspozarica.edu.mx (G.D.-A.); 2 Consejo Veracruzano de Investigacion Cientifica y Desarrollo Tecnologico (COVEICYDET), Av. Rafael Murillo Vidal No. 1735, Cuauhtemoc, Xalapa, Veracruz 91069, Mexico; 3 Facultad de Instrumentacion Electronica, Universidad Veracruzana, Cto. Gonzalo Aguirre Beltran S/N, Xalapa, Veracruz 91000, Mexico; 4 Tecnológico Nacional de Mexico/I.T. Puebla-División de Estudios de Posgrado e Investigación, Av. Tecnológico No. 420, Maravillas, Puebla 72220, Mexico; javier.floresme@correo.buap.mx; 5 Faculty of Electronics Science Meritorious, University Autonomous of Puebla, 4 Sur 104 Centro, Puebla 72590, Mexico; roberto.ambrosio@correo.buap.mx; 6 Electronics Department, National Institute for Astrophysics, Optics and Electronics, Sta. María Tonantzintla, Puebla 72840, Mexico; mmoreno@inaoep.mx; 7 Doctorado en Ciencias de la Ingenieria, Instituto Tecnológico Superior de Misantla, Km 1.8 Carretera Lomas del Cojolite, Misantla, Veracruz 93821, Mexico; cmahernandez@gmail.com

**Keywords:** high-DOF robot manipulator, industrial robot, path planning, multiple-query planner, resistive grid, obstacle avoidance, large sparse matrix

## Abstract

The applicability of the path planning strategy to robotic manipulators has been an exciting topic for researchers in the last few decades due to the large demand in the industrial sector and its enormous potential development for space, surgical, and pharmaceutical applications. The automation of high-degree-of-freedom (DOF) manipulator robots is a challenging task due to the high redundancy in the end-effector position. Additionally, in the presence of obstacles in the workspace, the task becomes even more complicated. Therefore, for decades, the most common method of integrating a manipulator in an industrial automated process has been the demonstration technique through human operator intervention. Although it is a simple strategy, some drawbacks must be considered: first, the path’s success, length, and execution time depend on operator experience; second, for a structured environment with few objects, the planning task is easy. However, for most typical industrial applications, the environments contain many obstacles, which poses challenges for planning a collision-free trajectory. In this paper, a multiple-query method capable of obtaining collision-free paths for high DOF manipulators with multiple surrounding obstacles is presented. The proposed method is inspired by the resistive grid-based planner method (RGBPM). Furthermore, several improvements are implemented to solve complex planning problems that cannot be handled by the original formulation. The most important features of the proposed planner are as follows: (1) the easy implementation of robotic manipulators with multiple degrees of freedom, (2) the ability to handle dozens of obstacles in the environment, (3) compatibility with various obstacle representations using mathematical models, (4) a new recycling of a previous simulation strategy to convert the RGBPM into a multiple-query planner, and (5) the capacity to handle large sparse matrices representing the configuration space. A numerical simulation was carried out to validate the proposed planning method’s effectiveness for manipulators with three, five, and six DOFs on environments with dozens of surrounding obstacles. The case study results show the applicability of the proposed novel strategy in quickly computing new collision-free paths using the first execution data. Each new query requires less than 0.2 s for a 3 DOF manipulator in a configuration space free-modeled by a 7291 × 7291 sparse matrix and less than 30 s for five and six DOF manipulators in a configuration space free-modeled by 313,958 × 313,958 and 204,087 × 204,087 sparse matrices, respectively. Finally, a simulation was conducted to validate the proposed multiple-query RGBPM planner’s efficacy in finding feasible paths without collision using a six-DOF manipulator (KUKA LBR iiwa 14R820) in a complex environment with dozens of surrounding obstacles.

## 1. Introduction

**Figure 1:**
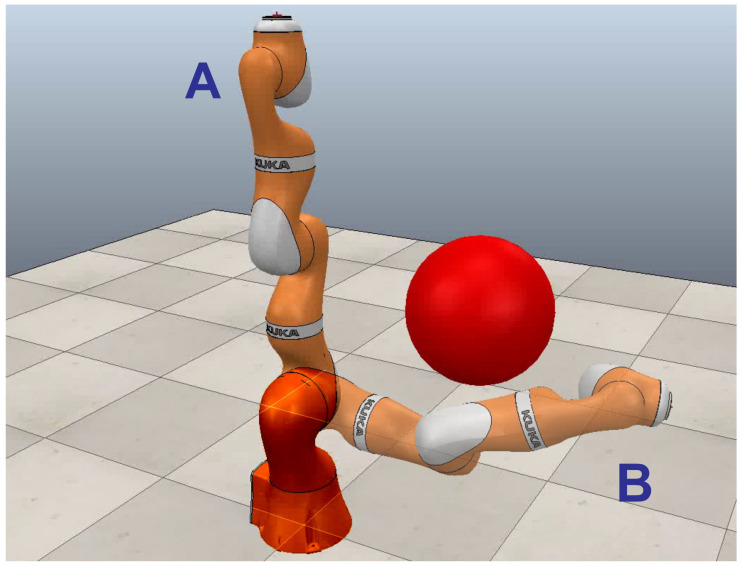
Robot manipulator collision-free path planning problem.

In various automated industry areas, robot manipulators are among the essential tools in a production line. These commonly work in static environments and are programmed to perform tasks in which they must move objects from one point to another within the workspace. That is, this process consists of taking the manipulator from configuration **A** to position **B**, as shown in Figure 1. Commonly, the path planning task is performed by an experienced robot programmer, who usually spends a considerable amount of time setting a sequence of manipulator movements that describes a collision-free path from **A** to **B**. The method most used for decades is based on demonstration; in this process, the programmer uses the robot’s teach pendant to move it from a collision-free configuration and gradually approaches the goal configuration [1,2]. These positions are saved in a sequence that is available to the robot to execute automatically on repeated occasions. This strategy is usually adequate in some approaches; however, it is impractical for many applications in which the human agent is not available or the task is too complex to be performed by a human. The importance of giving the robot the ability to independently calculate the sequence of the configurations would give it the flexibility to expand its applications and the skill to perform more than one task in a static workspace.

**Figure 2:**
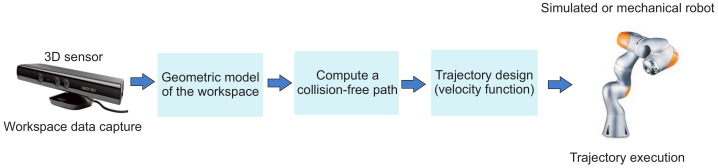
System architecture of an autonomous manipulator.

An autonomous robot is an intelligent agent capable of planning the configurations that it must run to perform a designated task. The success of this planning depends on the information the autonomous agent has about the environment. This information provides both the positions of the robot and the surrounding objects. Commonly, the information about the environment is provided by sensors onboard the robotic agent or by 3D cameras placed at strategic points in the robot’s workspace [3]. Figure 2 shows the basic architecture of an intelligent system based on a robotic manipulator. In this architecture, the environment data are provided through a 3D perception system to later obtain a geometric model compatible with the planning algorithm approach. The planning algorithm is in charge of calculating a feasible collision-free route usable by the manipulator. Before its execution, a control block must design a path as a function of velocity, considering the dynamic characteristics of the devices that comprise the manipulator. In principle, path planning for a robot (mobile robot or manipulator) is a geometric process in which its control module must find a collision-free path in its workspace. For this, the robot is considered a point in the configuration space ($C_{space}$), which is the space generated by all feasible positions that it can reach [4,5,6], where $C_{space}$ is divided into configuration space free ($C_{free}$) for valid positions and obstacles space ($C_{obs}$) for all forbidden configurations that imply a collision. The task of generating collision-free paths with applications in industrial robotics for manipulators is a field that has attracted the attention of many researchers due to the large demand in the market [7,8]. In this sense, numerous planning strategies have been developed with different approaches such as sampling-based algorithms [4,9,10,11], artificial potential fields [2,12], heuristic approaches [13,14,15], and grid-based planners [1,7,16,17,18]. The latter ones have properties that make them useful in industrial applications; the most relevant are the following: (1) The $C_{space}$ being generated only once for a static environment is the most common in industrial applications. (2) These are considered complete resolution planners, i.e., if a solution exists, any of these algorithms can obtain one; otherwise, the method terminates and reports that no solution exists for the specified resolution [4]. (3) The representation of the obstacles is straightforward since occupation cells’ representation is used. (4) The $C_{space}$ is easily stored in a bitmap.

Commonly, the path planning methods only focus on the end-effector position; however, all the joints need to be considered during the movement. In this case, the inverse kinematics problem is also a key theme for path planning. Many strategies and algorithms of path planning for a manipulator have been proposed in the literature. These works mainly focused on the two aspects: reactive planning and map-based planning [19,20]. For reactive planning, the robot has a perception system that allows it to know the environment in which it performs its task, and its main application is for environments with dynamic obstacles. For map-based planning, an RGB-D camera or 3D laser sensor provides the map i.e., the workspace is known a priori and obstacles are considered static objects [3]. For the reactive planning approach, a smoothly RRT (S-RRT) algorithm was proposed [21]. This work focused on obtaining smoother paths using S-RRT, and its effectiveness was validated through a case study of one static and one dynamic obstacle. Using the same approach, ref. [3] proposed an algorithm to obtain a 3D workspace representation and compute a reorientation object path using a manipulator. For map-based planners, the following works can be found: First, for low-DOF and low obstacles density [22], employed a path planner based on a multi-resolution potential field to calculate collision-free paths for a four-DOF planar manipulator in 2D workspaces. Similar to previous work, in [23], a resistive grid-based path planning method is proposed to find collision-free trajectories in 2D workspaces for a three-DOF planar manipulator with few obstacles (maximum of five obstacles). A novel grid-based approach for 3D workspaces with three static obstacles and a manipulator of three DOFs is presented in [24]. Second, for high DOFs with a low obstacle density, an intermediate point obstacle avoidance algorithm for a 6 DOF serial manipulator and two static obstacles is presented in [25]. Similarly, an algorithm based on the artificial potential field was reported [26], where the analysis focused on a 6 DOF industrial manipulator over a workspace with few obstacles. Finally [27], reported a powerful obstacle avoidance path planning method, using forward- and backward-reaching inverse kinematics to compute a solution path of a seven-DOF manipulator for a 3D workspace with three static obstacles. Most of the work in the literature has only obtained collision-free trajectories considering fewer than five obstacles in the environment and fewer than four joints. These methods are often fast and efficient for real industrial applications. However, a planner capable of handling complex environments with a high density of obstacles and high-DOF manipulators is essential for most of the industrial sectors’ automated processes. Another important fact to consider is that approaches based on artificial potential fields and RRT require a reprocessing of the entire configuration space for each query. However, for grid-based approaches and some probabilistic road-map-based planners, the path recalculation is less computationally expensive using the first simulation data. In this sense, grid-based planners possess multiple properties that make them suitable for implementation in intelligent robots for industrial applications.

### Grid-Based Planning Algorithms

Grid-based planners (GBPs) contain all discrete-space-based algorithms; generally, these are the best choice for obtaining a valid path for maze-type environments. However, for non-structured environments, its performance depends on the resolution of the grid [4,28]. These planners commonly operate in two stages: (1) generation of $C_{space}$ and (2) search for the solution path. In the first stage, these planners create a map of the $C_{space}$, which is divided into configuration space free ($C_{free}$) for valid positions of the robot and obstacles space ($C_{obs}$) for all forbidden configurations that imply a collision. This configuration map is stored in an *n*-dimensional bit array, where *n* is the number of degrees of freedom (DOFs) of the manipulator, and each dimension of the array is of size $s_{n}$. In this map, each of the bits corresponds to the manipulator’s configuration, and free configurations and prohibited configurations must be distinguished. In the second stage, a search algorithm (such as $A^{*}$, Dijkstra’s, or local current comparison) is used to find the solution path represented by a sequence of valid configurations in $C_{free}$ that lead from configuration **A** to configuration **B** [4,11,23,28].

The GBPs have some important properties that make them useful in industrial applications such as (1) the generation of the $C_{space}$ is carried out only once for a static environment, (2) these are considered resolution complete planners, (3) the representation of the obstacles is straightforward since the representation by occupation cells is used, and (4) the $C_{space}$ is easily stored in a bitmap of shape $\left(k_{1}+1\right)\times \left(k_{2}+1\right)\times \left(k_{3}+1\right)\times |\ldots \times \left(k_{n}+1\right)$, where *n* is the number of the manipulator’s joints, $\left(k_{i}+1\right)$ is the number of possible values of each joint angle, and $k_{i}$ is the size of the grid of the *i*th joint. Recently, one of the planning methods that was proven to be efficient and versatile in intelligent system applications is the resistive grid-based planner [16,23,29,30,31]. It has all the features described above that commonly distinguish a grid-based planner. Additionally, its operation is based on the physical principle of current flow from a source (start point) to a potential sink (target point), where the solution path (from start to target both configurations of $C_{free}$) is described by the path of least resistance within a network for which each node in it corresponds to a configuration in $C_{free}$.

In this work, a multiple-query planner inspired on the resistive grid-based method was constructed. The proposed method is capable of calculating collision-free paths for manipulators with high DOFs for applications in complex industrial environments. This paper is organized as follows: In Section 2, the bases of the resistive grid-based planning method are explained. The resistive grid-based planning method applied to industrial robot manipulators is presented in Section 3. A multiple-query approach of the resistive planner is explained in Section 4. Three case studies of path planning examples applying the multiple-query RGBPM are provided in Section 5. Finally, the conclusions and future work are described in Section 6.

## 2. Resistive Grid-Based Planning Method

**Figure 3:**
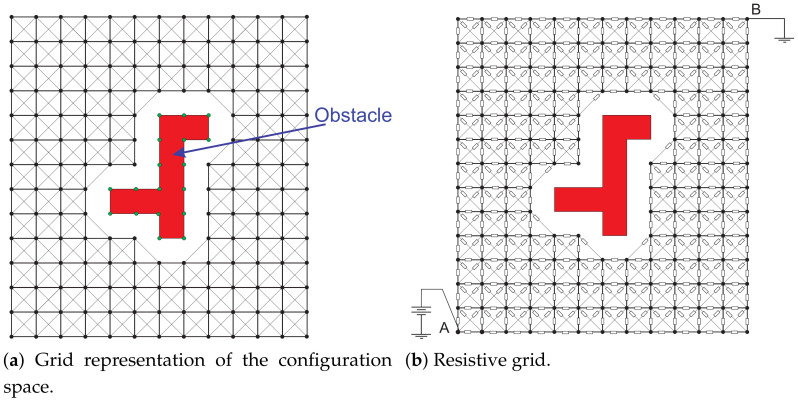
Two-dimensional resistive grid representation of the configuration space.

The resistive grid-based planning method operates in two stages, similar to other grid-based planners. In the first stage, a continuous $C_{space}$ is discretized into a mesh of resolution *k*, where $C_{free}$ is composed by all cells that are not occupied by an obstacle. Figure 3a shows a discretized 2D workspace in which a guard distance is considered between the obstacle and nodes of $C_{free}$ to reduce the collision risk. For the resistive grid-based planning method, the $C_{space}$ is modeled using a mesh of resistances, where each node is associated with a $C_{free}$ configuration, and each resistor represents the movement required to proceed from one configuration to another. The resistive grid’s geometry determines the position of each node in the $C_{space}$; it has a direct influence on the resolution of the motion, which determines the nature and smoothness of the manipulator’s movements. In this work, the implementations are performed using a square geometry because its implementation in multidimensional spaces is straightforward [16]. The square mesh provides diagonal paths along with horizontal and vertical paths, as depicted in Figure 3a. The second stage (search solution path) is performed through the local current comparison algorithm (LCC). The LCC operates as follows: (1) a voltage source is connected to the start configuration A and the ground reference at B, as shown in Figure 3b; (2) the instantaneous current flows from A to B, where the optimal solution path (shortest) is denoted as the least resistance path, i.e., the path of the larger current flow [29,30,31,32].

**Figure 4:**
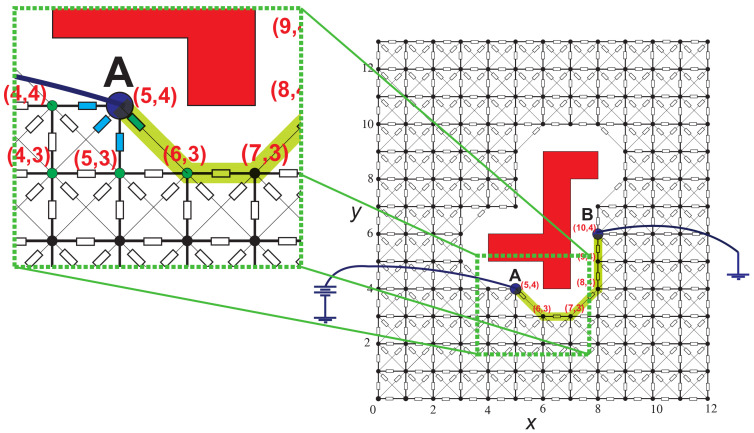
Local current comparison representation.

In principle, the LCC algorithm is a sequential procedure in which the less resistive path from the power supply to the GND is found from the voltage nodal values of all the nodes in $C_{free}$. Figure 4 shows the solution path from **A** to **B** and the LCC procedure. The LCC process begins at node **A** connected to the power supply and ends when a connection is created to the node connected to the GND. A closeup view of the image segment shows the analysis to follow, where from node **A** (positioned at coordinates (5,4)), the next segment of the path with the greatest current is determined employing a local comparison with each of the neighbors (nodes: (4,4), (4,3), (5,3), (6,3)). Notably, the LCC algorithm depends on the prior knowledge of the electrical variables’ values and all resistors in the resistive network. The value of each resistance is obtained from the grid’s geometric distribution, where, for each resistor, the value is set proportional to the Euclidean distance between the nodes to which it is connected [29]. However, to know the electrical variables’ values, a circuit analysis tool must be used that calculates the operating point of the resistive network.

The formulation and solution of a linear system of KVL are standardized tasks through nodal analysis (NA), and modified nodal analysis (MNA), the latter of which is an industrial standard for circuit simulation programs [23,30,33]. In principle, for an original formulation, only two stamps are required to formulate the RG circuit’s equilibrium equation. First, the conductance stamp is:

(1) $$\begin{array}{cccc} & v_{i} & v_{j} & \mathrm{RHS}\\ i & G & -G & 0\\ j & -G & G & 0 \end{array}$$

where $v_{i}$ and $v_{j}$ represent each nodal voltage, *i* and *j* are the nodes of the component (the position of the node in the $C_{space}$), G=1/R is the equivalent conductance, and RHS is the right-hand side of the equilibrium equation. We used the equivalent conductance (1/R) because its stamp is simpler than the resistance’s stamp, and it does not add extra variables to the equilibrium equation [29].

Second, the stamp of the independent voltage sources (IVS is):

(2) $$\begin{array}{cccc} & v_{i} & i_{E} & \mathrm{RHS}\\ i & 0 & +1 & 0\\ \mathrm{BE} & +1 & 0 & E \end{array}$$

where $v_{i}$ represents the nodal voltages, *i* is the terminal node of the IVS in the RG, *E* is the voltage supplied by the IVS, and $i_{E}$ is the unknown current that crosses the IVS. Notably, the IVS adding an extra column corresponds to $i_{E}$, and an extra row corresponds to the branch equations (BEs) of the IVS [16,23]. In addition, a characteristic to note in the RGBPM method is that a defined grid in 2D of $s_{1}\times s_{2}$ generates a linear system of MNA equations of ${\left(s_{1}\times s_{2}\right)}^{2}+1$. In this sense, an *n*-dimensional system produces a sparse matrix of size ${\left(\left(s_{1}\times s_{2}\times \ldots \times s_{n}\right)+1\right)}^{2}$.

## 3. RGBPM Applied to Industrial Robot Manipulators

**Figure 5:**
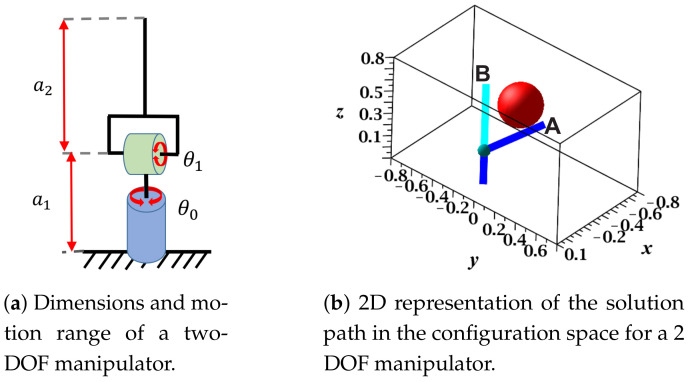
Dimensions, motion range, and start–target configurations of a two-DOF manipulator.

RGBPM is a versatile tool that has been used to find collision-free paths for mobile robots [30,31,32], as a technique to locate the shortest route in vehicle guidance applications [29] and collision-free path planning for planar manipulators [16,34]. Although this versatility has only been exploited for two- and three-dimensional grids, for high-dimensionality, the complexity of representation is a difficult task as the current RGBPM implementations in [16,23,34] do not report any methodology capable of providing a suitable model or mathematical representation. This section explains how the implementation of RGBPM is possible in real industrial manipulator applications and the restrictions that must be considered for its correct implementation. A representation of the configuration space through proper and efficient obstacles models is presented. This section and the subsequent ones represent the most notable contributions of this work. To explain the formulation and obtaining of the $C_{space}$ as well as the $C_{free}$, the 2 DOF model is shown in Figure 5a. Table 1 contains the Denavit–Hartengberg (D-H) parameters of the two-DOF manipulator with their respective angle ranges and lengths of links. Additionally, Figure 5b shows the planning used to solve where the start configuration is marked with **A** and the target position **B**. The environment is a simple problem with only one spherical obstacle that interposes between the manipulator’s configurations.

### 3.1. Creation of Discrete Configuration Space

**Figure 6:**
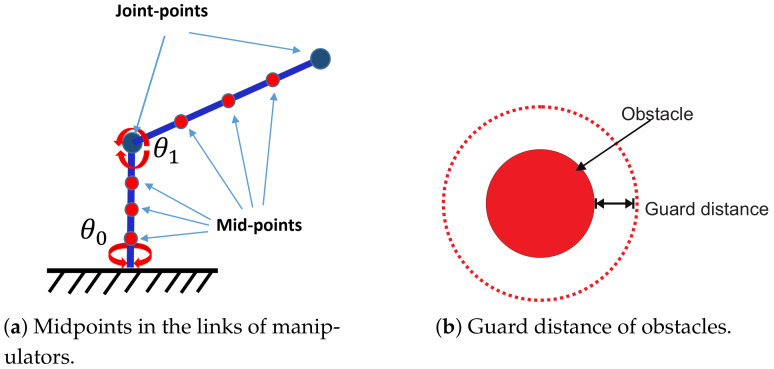
Midpoints and guard distance of obstacles.

An adequate description of the configuration space is the key to finding a solution path for robotic manipulators. For these types of applications, only the end-effector position is commonly considered; however, a collision-free path can only be obtained if all the manipulator links are considered in the process. For this, a $C_{espace}$ formulation that considers both manipulator models and obstacles and guarantees no collisions is necessary. This subsection presents obstacles representations through closed surfaces using spheres, toroids, and limits through mathematical expressions. For a manipulator, the $C_{space}$ is a set of all positions that it can reach. This space is composed by two subsets, $C_{free}$ and $C_{obs}$, where the first contains all the collision-free configurations that the manipulator can execute and the second contains the forbidden configurations that represent the space occupied by obstacles in the workspace. In this work, a square geometry grid similar to that presented in [16,34] was employed. Additionally, the following characteristics were considered to generate a path planner focused on industrial robots with representation in real workspaces. The considerations are as follows:

- A set of midpoints are established to obtain a representation of solid links. The number of intermediate points is selected according to the criteria presented in [35]. The distance between two intermediate points (considering the junction points) must not be greater than the minimum size of an obstacle so that there are no collisions with small obstacles.
- A guard distance is established as the distance between the central axis of each link with its external reinforcement; thus, each link can be considered as a line segment similar to the one presented in Figure 6a. The guard distance is determined according to the dimensions and morphology of the manipulator used. Figure 6b shows a circular obstacle in 2D with a guard distance added.
- The obstacles and limits in 3D space are produced by geometrical representation using closed curves and primitive representations [4]. For this work, the shapes of obstacles are spheres, denoted by Equation (3), and torus, denoted by one of the expressions in (4).
  (3) $$S={\left(x_{i}-x_{c}\right)}^{2}+{\left(y_{i}-y_{c}\right)}^{2}+{\left(z_{i}-z_{c}\right)}^{2}-r^{2},$$
  (4) $$\begin{array}{c} T_{x}={\left(R_{t}-\sqrt{{\left(y-y_{c}\right)}^{2}+{\left(z-z_{c}\right)}^{2}}\right)}^{2}+{\left(x-x_{c}\right)}^{2}-r_{t}^{2},\\ T_{y}={\left(R_{t}-\sqrt{{\left(x-x_{c}\right)}^{2}+{\left(z-z_{c}\right)}^{2}}\right)}^{2}+{\left(y-y_{c}\right)}^{2}-r_{t}^{2},\\ T_{z}={\left(R_{t}-\sqrt{{\left(x-x_{c}\right)}^{2}+{\left(y-y_{c}\right)}^{2}}\right)}^{2}+{\left(z-z_{c}\right)}^{2}-r_{t}^{2}. \end{array}$$
  The limits of each dimension in 3D space are delimited by primitive expression:
  (5) $$L=|v_{i}-l_{v}|\pm \left(v_{i}-l_{v}\right),$$
  where $v_{i}$ is the value of the limit on the *v* axis. Using this representation, a 3D box can be generated in the workspace that restricts the manipulator’s movements and limits the total valid configurations in $C_{space}$.
- The mathematical model to obtain a bitmap of $C_{free}$ evaluates the position of each midpoint and joint point for a configuration of joint angles θ in the obstacles expression (6), where if $C_{obs}>0$, then the position of the determined θ configuration is equal to 1 (which implies a collision-free configuration).
  (6) $$C_{obs}=\prod_{i=1}^{n}\left(\right|O_{i}\left|+\left(O_{i}\right)\right),$$
  where $|O_{i}|$ is the absolute value of $O_{i}$, where $O_{i}$ represents the algebraic representation of the *i*th obstacle. The obstacles can be spherical denoted by Expression (3), torus denoted by Expression (4), or limit denoted by Expression (5).
- The position $\left(x_{i},y_{i},z_{i}\right)$ of each intermediate point and junction point in the workspace is determined by formulating quaternions and using Denavit–Hartenberg parameters, where the position of each *i*th midpoint and joint point is:
  (7) $$\vec{P_{i}}=A_{0}\cdot A_{1}\cdot \ldots \cdot A_{i-1}\cdot \vec{P_{0}},$$
  where $A_{j};\;j=0,1,2,\ldots i-1$ is a homogeneous matrix calculated from the D-H parameters and $\vec{P_{0}}$ is the vector that represents the origin of the transformation system for each midpoint or joint-point.
  (8) $$\vec{A_{j}}=R_{z,\theta_{j-1}}\cdot T_{z,d_{j}}\cdot T_{x,c_{j}}\cdot R_{x,\alpha_{j-1}},$$
  where *R* represents the rotation matrix with respect to the variable *v* for v=x,z, the joint angle is represented by $\theta_{j}$, and $\theta_{j}$ represents the twist angle for the *j*th link. $c_{j}$ is the length of the *j*th link, and $d_{j}$ is the offset of linkages for the *j*th link.

**Figure 7:**
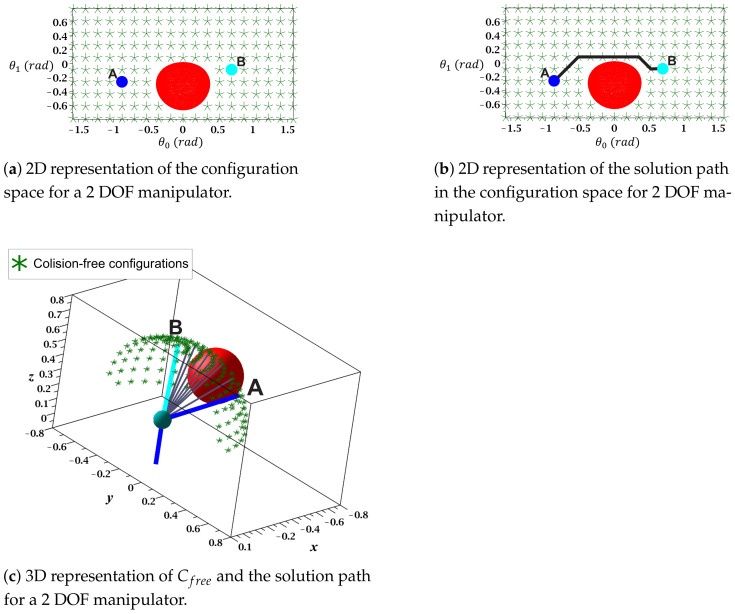
Configuration space ($\theta_{0}-\theta_{1}$) for a 2 DOF manipulator.

Figure 7a shows a 2D representation for the motion angles of each junction $\left(\theta_{0},\theta_{1}\right)$, where the $C_{space}$ is discretized for a cell size of $10^{{}^{\circ}}$ in both axes. This figure also shows the angle combinations that produce collision-free configurations, which are marked with asterisks. To obtain the 2D map in $C_{space}$, a set of 20 midpoints was employed for each link, and the obstacles representation was obtained from (6) for $C_{obs}=0$. Additionally, a guard distance of 0.01 m was considered. Figure 7b shows the solution path from **A** to **B** formed by a set of collision-free configurations. This figure also shows that the route is optimal (in terms of length), and each configuration is collision-free due to the established guard distance. The representation and execution of the solution path displayed on the 3D workspace performed on MapleSoft is shown in Figure 7c. This figure also shows the sequence of movements that the manipulator followed to reach **B** in grey.

## 4. A Multiple-Query RGBPM

Multiple-query planners are potent tools that provide an intelligent agent with the ability to develop more than one task in a static workspace. Commonly, the manipulators perform a designed work in industrial workspaces, which are usually static environments. A multiple-query planner can provide the robot the skill to perform two or more activities on the same production line without depending on an expert operator to configure each of the routes. For RGBPM, a multiple-query ability implies that the source and ground connection nodes must be changed in each query, while the resistive grid’s arrangement must not have any change. If the original formulation of RGBPM is used, the change in the destination node does not alter the formulation; however, a change in the position of the voltage source alters the entire configuration of the MNA matrix, i.e., the solution of the linear system for each query will be different [16,23,30,32,36]. To solve these issues, three changes are proposed here that improve the performance of the RGBPM and result in a novel multiple-query planner. The improvements are as follows:

1. The voltage source of the start configuration and the GND of the target are replaced by two independent current sources (CSs): one with a positive value and the second with a negative value, respectively. The stamp to integrate the CS in the MNA formulation is:
  (9) $$\begin{array}{ccc} & v_{i} & \mathrm{RHS}\\ i & 0 & J \end{array}$$
  where *J* is the value of the CS connected at node *i*. Importantly, only two CS elements are required for the new implementation, and the CS integration does not modify the MNA matrix.
2. A dummy resistor with a large value is placed between each *i*th node in the resistive grid and GND. This strategy guarantees that the MNA matrix in the formulation is never singular. The stamp of each dummy resistor is represented by:
  (10) $$\begin{array}{ccc} & v_{i} & \mathrm{RHS}\\ i & G_{d} & 0 \end{array}$$
  where $G_{d}=1/\left(R_{d}\right)$. Then, for the experiments and implementations in this work, $R_{d}=1\times 10^{9}$.
3. Using the two previous changes in the formulation of the equilibrium equations of the resistive network, the system then takes the following form:
  (11) Ax=b,
  where *A* is an (m×m) matrix that contains all the values of the resistors in the RG (including all dummy resistors); *x* is the vector of nodal voltages, each one associated with one node in $C_{free}$; and *b* is the incidence vector that contains the values of the two CSs. The importance of the changes to the formulation can be seen more clearly in this expression since now, if the position of the sources (CSs) changes, the effect is only reflected in vector *b*, and matrix *A* does not undergo any change. Now, the multiple-query problem becomes the task of solving linear systems in which the vector *b* changes for each query. As mentioned previously, the arrangement of resistors in RG produces very large sparse matrices, where the size of the matrix is determined by the number of nodes (*m*) in $C_{free}$. In this work, the incomplete LU factorization (ILU) is used to obtain a reliable approximation of the solution with a reduced processing time. The ILU was selected due being one of the most common approaches to solving large sparse linear systems [37,38,39,40]. The ILU factorization operates as follows:
  (12) $$Ax=b\to M^{-1}Ax=M^{-1}b,$$
  where $M^{-1}$ is the approximate inverse matrix and the solution is obtained through $M^{-1}b$ calculation. In this approach, M=LU for *L* and *U* is the approximation of lower and upper triangular matrices, respectively [39]. In this sense, we verified that using a first execution of the ILU method, the inverse matrix $M^{-1}$ can be recycled for subsequent changes in the vector *b*, thus reducing the processing time of each query in RGBPM.

**Figure 8:**
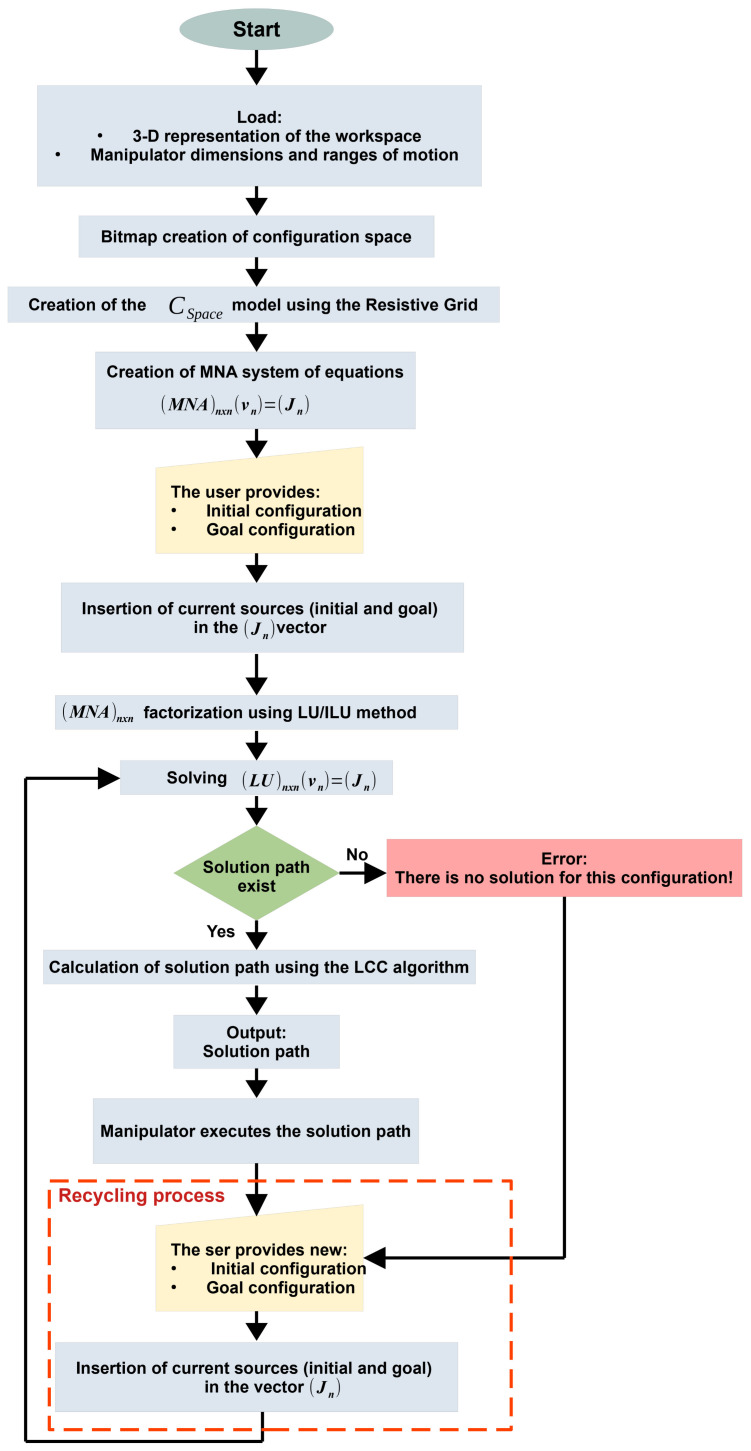
Flowchart of multiple-query RGPM for high-DOF manipulators.

Figure 8 depicts the complete flowchart of the proposed multiple-query RGBPM. This flowchart shows that the inputs of the planner are the 3D representation of the workspace considering all obstacles (positions, dimensions, and shapes) and the manipulator dimensions, motion range, and the grid resolution in terms of steps of the motion angle of each joint. The path is provided if a solution exists for the configuration and the resolution of the grid selected. The processes associated with the multiple-query skill are marked as a recycling process, where they generate a loop with the first execution results. The most relevant and non-trivial RGBPM processes are explained in Algorithms 1–3.

First, Algorithm 1 generates a bitmap object from robot and workspace specifications. This is a process of *n* for-loops nested, where *n* is the number of DOFs of the manipulator that determines the number of for-loops in the process. A valid configuration is denoted as 1 in the bitmap when the evaluation of such angles in the $C_{obs}$ is greater than 0. This denotes that the core of this process is evaluating the set of values of the angles of movement of each joint in the expression $C_{obs}$.
**Algorithm 1** Bitmap creation of the configuration space.  1:**procedure**Bitmap(${\mathrm{Specs}}_{obstacles},{\mathrm{Specs}}_{manipulator}$) ▷ Specifications of the manipulator are denoted by D-H parameters  2:   3: **for** $\left(i_{0}=0:i_{0}\leq \left(\theta_{range}^{i_{0}}\right)/\left(S_{size}^{i_{0}}\right):i_{0}++\right)$ **do**  4:  ⋮  5:  **for** $\left(i_{n-1}=0:i_{n-1}\leq \left(\theta_{range}^{i_{n-1}}\right)/\left(S_{size}^{i_{n-1}}\right):i_{n-1}++\right)$ **do**   ▷ *n* is the number of DOFs of the manipulator  6:   7:   **if** $\left(\mathrm{eval}(C_{obs},\left[\theta_{0},\cdots ,\theta_{n-1}\right])>0.0\right)$ **then** ▷ Evaluation of the obstacle model to determine if there is a collision.  8:    Bitmap$[i_{0},\cdots ,i_{n-1}]=1$  9:   **end if**10:  **end for**11: **end for**12: **return** [Bitmap]13:**end procedure**

Second, Algorithm 2 is the process through which the MNA matrix is obtained from the bitmap using the stamps formulation of the current sources and of all resistors (resistors that connect each pair of $C_{free}$ nodes and dummy resistors) in the RG. The size of the MNA array corresponds to the number 1s in the bitmap, i.e., the number of collision-free nodes in $C_{space}$. For this procedure, the core of the process is the addition of each resistance’s value in the elements of the MNA matrix according to the corresponding stamp for the positions of each pair of nodes.
**Algorithm 2** Resistive grid and MNA matrix creation.  1:**procedure** RG(Bitmap)  2: $R_{u}=1$                     ▷ Unit value resistor  3: $R_{dummy}=1\times 10^{10}$           ▷ Very large dummy resistor value  4: **for** $\left(i_{0}=0:i_{0}\leq \left(\theta_{range}^{i_{0}}\right)/\left(S_{size}^{i_{0}}\right):i_{0}++\right)$ **do**  5:  ⋮  6:  **for** $\left(i_{n-1}=0:i_{n-1}\leq \left(\theta_{range}^{i_{n-1}}\right)/\left(S_{size}^{i_{n-1}}\right):i_{n-1}++\right)$**do**   ▷ *n* is the number of DOFs of the manipulator  7:   8:   $N_{current}=\mathrm{Bitmap}\left[i_{0},\cdots ,i_{n-1}\right]$  9:   **for** $\left(j=0:j\leq (k_{neighbor}-1):j++\right)$**do**   ▷ Number of neighbor nodes $k_{neighbor}$ depends on the DOFs of the $C_{space}$10:    **if** $\left(N_{current}==1\right)$ **then**11:     $\mathrm{add}\_\mathrm{Node}({\mathrm{Nods}}_{list},N_{current})$   ▷ Current node ($N_{current}$) is added to the node list12:     $Node_{1}=N_{current}$13:     $\mathrm{add}\_\mathrm{MNA}\left(\mathrm{Pos}\left(Node_{1}\right),R_{dummy}\right)$    ▷ Resistors related at node position are added to the MNA matrix14:     **if** $\left(N_{neighbor}\left[j\right]==1\right)$**then**   ▷ Determines if a neighbor node is a valid configuration15:      $Node_{2}=N_{neighbor}\left[j\right]$16:      $R_{m}=R_{u}/\left(∥Node_{1}-Node_{2}∥\right)$17:      $\mathrm{add}\_\mathrm{MNA}\left(\mathrm{Pos}\left(Node_{1}\right),\mathrm{Pos}\left(Node_{2}\right),R_{m}\right)$18:     **end if**19:    **end if**20:   **end for**21:  **end for**22: **end for**23: **return** $\left[{\mathrm{Nods}}_{list},\;\mathrm{MNA}\right]$24:**end procedure**

Finally, the LCC depicted in Algorithm 3 is a sequential procedure that, together with the values obtained from the solution of the linear system using ILU factorization, operates as a search algorithm. It iteratively determines the path of least resistance by comparing the branch currents between a node and its neighbors. In this way, this process is followed until reaching the target point. For this process, the number of comparisons in each search step is $3^{n}-1$, where *n* is the number of motion angles of the manipulator.
**Algorithm 3** Calculation of the solution path using LCC.  1:**procedure** LCC($Node_{voltages}$, bitmap, $Node_{start}$, $Node_{target}$)  2: path=[ ]  3: $R_{u}=1$                     ▷ Unit value resistor  4: $Node_{test}=Node_{start}$  5: $Max_{current}=-1\times 10^{6}$  6: j=1  7: $\mathrm{path}=Node_{start}$  8: **while** $\left(Node_{test}\neq Node_{target}\;\mathrm{and}\;j\leq Max_{steps}\right)$ **do**  9:  $Nodes_{neighbor}=\mathrm{Neighbors}\left(Node_{test}\right)$10:  **for** $\left(k=1:k\leq \mathrm{elements}\left(Nodes_{neighbor}\right):k++\right)$**do**    ▷ The number of $Nodes_{neighbor}$ varies according to the DOF of manipulator11:   **if** ($\mathrm{Bitmap}[\mathrm{Pos}\left(Node_{neighbor}\left[k\right]\right)]=1)]$) **then**12:    $R_{m}=R_{u}/\left(∥Node_{test}-Node_{neighbor}\left[k\right]∥\right)$13:    $I_{k}=\left|Node_{voltages}\left[\mathrm{Pos}\left(Node_{test}\right)\right]-Node_{voltages}\left[\mathrm{Pos}\left(Node_{neighbor}\left[k\right]\right)\right]\right|/R_{m}$14:    **if** $I_{k}>Max_{current}$ **then**   ▷ The path of greatest current flow between the test node ($Node_{test}$) and neighboring nodes is determined15:     $Max_{current}=I_{k}$16:     $Node_{next}=Node_{neighbor}\left[k\right]$17:    **end if**18:   **end if**19:  **end for**20:  $\mathrm{path}=\mathrm{path},Node_{next}$ ▷ The node with the greatest current flow is added to the path21: **end while**22: **return** [path]23:**end procedure**

## 5. Case Studies

This section describes three case studies used to explain the usefulness of the proposed methodology, how it solves path planning problems for static workspaces with dozens of obstacles, and its powerful ability to rapidly compute new paths using the ILU factorization of the MNA matrix from the first execution. The two first cases focused on applying the proposed strategy to calculate a collision-free path for redundant manipulators visualized by a model performed in MapleSoft. Case study three demonstrates the effectiveness of RGBPM in finding a collision-free path for a standard industrial manipulator visualized by a model in a V-REP simulator (KUKA LBR iiwa 14R820). All simulations were performed using the following set-up: our proposed multiple-query RGBPM was coded in Python 3.8 with sparse-matrix solvers packages; each matrix was stored on a csc_matrix format and executed on PC with an Intel i7 10 processor @ 2.6 GHz, RAM 64 GB, and 64 bit Windows 10 operating system.

### 5.1. Case 1: 3 DOF Robot Manipulator (Visualization on MapleSoft)

**Figure 9:**
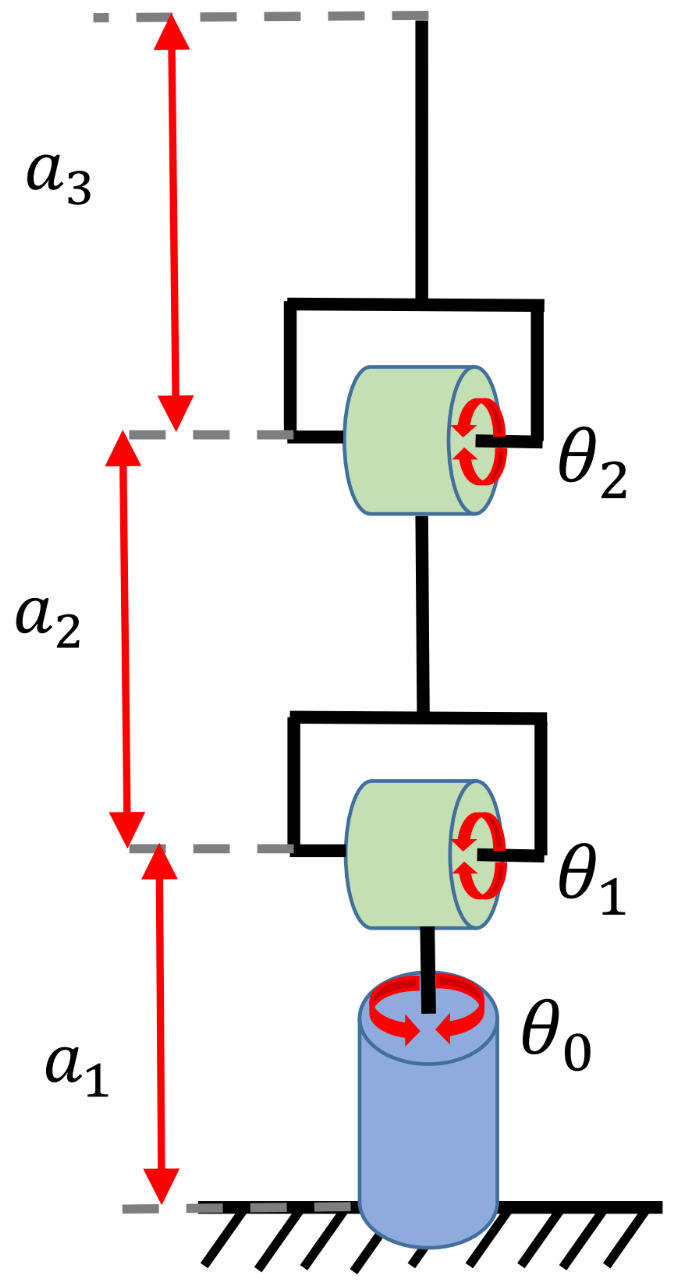
Dimensions and motion range of a 3 DOF manipulator.

**Figure 10:**
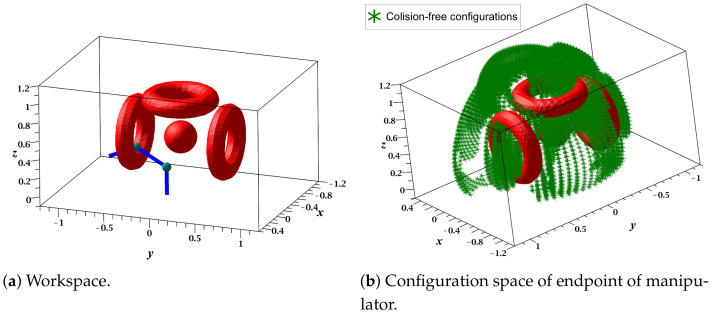
Workspace and Configuration space (3 DOF manipulator).

This case study focused on demonstrating the ability of the proposed RGBPM to handle spaces with torus and solid spherical obstacles using the representation in Section 3.1. The configuration of the manipulator is depicted in Figure 9 and its D-H parameters, motion range, and dimensions are listed in Table 2. For this study case, the resolution of each joint angle was $5^{{}^{\circ}}$, then the bitmap of the $C_{space}$ was an array of shape 37×19×37, i.e., a total of 26,011 valid configurations of the manipulator (considering $C_{free}$ and $C_{obs}$ configurations). The visualization of this manipulator was performed on MapleSoft (see Figure 10a); for this visualization and implementation, each link was considered as a solid line using 20 midpoints. Furthermore, we considered a guard distance in the size of each obstacle. Using the dimensions of each link, the range of motion angles, and the mathematical representation of each obstacle (torus and spherical obstacles), the endpoint’s possible configurations were obtained, as shown in Figure 10b. This representation shows the points achievable in an industrial application in which the endpoint contains the end effector (gripper, welder, or some other tool).

**Figure 11:**
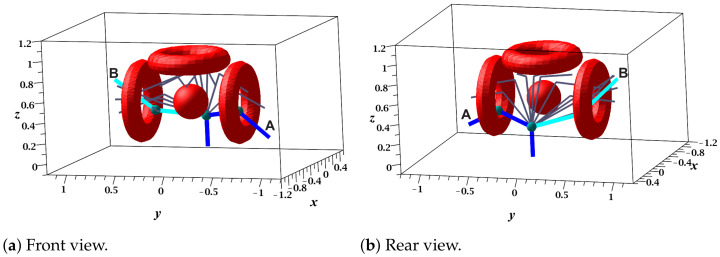
A 3 DOF manipulator collision-free path of query 1 (sequence of configurations): $(A)=\left(60^{{}^{\circ}},10^{{}^{\circ}},-80^{{}^{\circ}}\right);\;\left(B\right)=\left(-60^{{}^{\circ}},-35^{{}^{\circ}},-30^{{}^{\circ}}\right)$.

Using a grid size of $5^{{}^{\circ}}$ as well as the workspace specifications, the RGBPM was able to determine that the $C_{free}$ consisted of a total of 7291 configurations. These free space configurations correspond to the total number of nodes of the resistive network (with a density of 59,953 resistors), which model the $C_{free}$ grid. Table 3 shows the main results obtained in the first execution of the RGBPM (Figure 11a,b front and rear views, respectively). The bitmap was created in 11.44285 s, while each one of the central iterations in which whether a collision exists or not was calculated consumed between 0 and 0.0009982 s, which are the best and the worst times, respectively. The variation in this result depended on the position of the midpoint for which the collision was detected, being the shortest time if the collision was detected in the first midpoint (time very close to zero, being so small that it was not possible to measure it) or detected at the last manipulator midpoint (considering all manipulator midpoints and junction points). The total time for the generation of the equilibrium equations system using the MNA formulation was 6.2851 s. Here, each iteration of this process required between 0 and 0.001994 s; the time varied depending on how many neighboring nodes equal to 1 existed; the more the collision-free neighboring nodes, the larger the number of resistances; therefore, a greater number of elements must be added to the MNA formulation.

**Figure 12:**
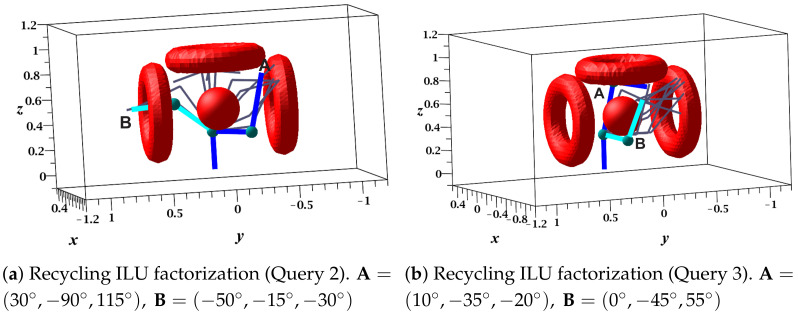
Recycled collision-free paths of the 3 DOF manipulator.

Once the bitmap is generated and MNA formulation and LU factorization processes are executed, the RGBPM is able to recycle the results to obtain infinite solution paths for any start and end nodes as long as they are in the list of collision-free nodes. Figure 12a shows the sequence of configurations (solution path) for the start (A) and end (B) positions established for the movement angle configurations $\left(60^{{}^{\circ}},10^{{}^{\circ}},-80^{{}^{\circ}}\right)$ and $\left(-60^{{}^{\circ}},-35^{{}^{\circ}},-30^{{}^{\circ}}\right)$. In the same way, the sequence for the query 3 from point $A=(60^{{}^{\circ}},10^{{}^{\circ}},-80^{{}^{\circ}})$ to $B=(-60^{{}^{\circ}},-35^{{}^{\circ}},-30^{{}^{\circ}})$ is presented in Figure 12b.

For each of these queries, two drop tolerance criteria were used for ILU factorization (α: ILU-tolerance = $1\times 10^{-3}$, β: ILU-tolerance = $1\times 10^{-6}$) in order to measure the impact of this parameter on the length and execution time for each query. The results are presented in Table 4, where the following can be denoted: (1) The execution times of the ILU factorization for both criteria were less than 0.05 s (this time is only considered in query 1), while the resolution of the system using the factoring results required less than 0.002 s for the three queries using both tolerance criteria. (2) A smaller tolerance criterion did not always imply a shorter path; however, it was guaranteed to be more successful when finding a solution path. The above can be verified by the results of query 2, in which for the α criterion, it is not possible to calculate a solution, but it is possible to calculate a solution when using the β criterion. Notably, each of the solutions is only an approximation of the real value, and therefore, the path will present many variations in terms of the length regardless of whether α is greater than β.

### 5.2. Case 2: 5 DOF Robot Manipulator (Visualization on MapleSoft)

**Figure 13:**
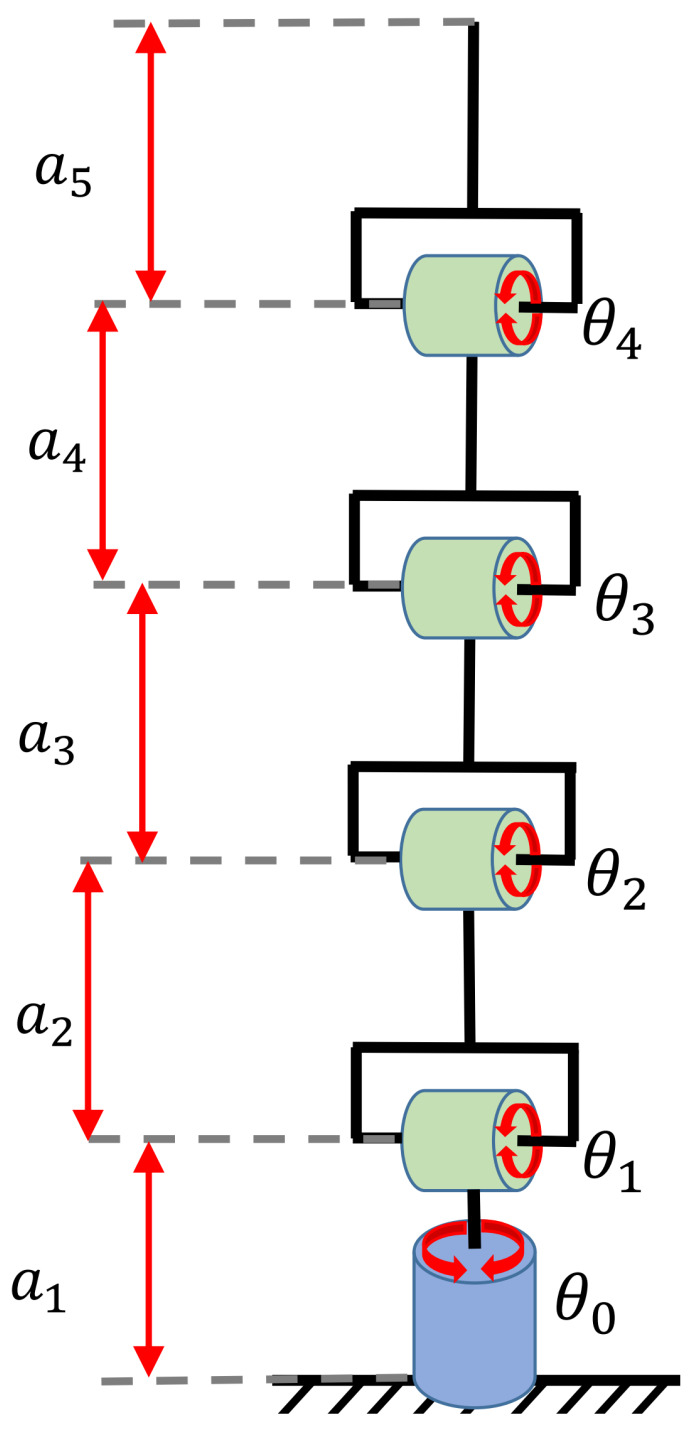
Dimensions and motion range of the five DOF manipulator.

**Figure 14:**
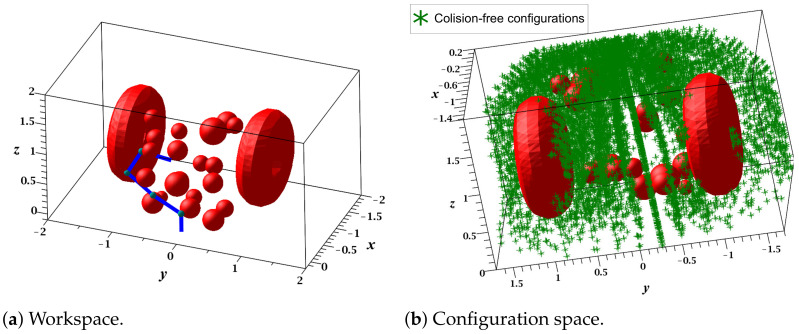
Workspace and configuration space of the five-DOF manipulator.

Similar to the previous case study, this experiment focused on exploring the ability of RGBPM to handle spaces with torus and solid spherical obstacles. Figure 13 presents the configuration of the five DOF manipulator. The D-H parameters, dimensions of links, and the motion range of joint angles are provided in Table 5. For this study case, the resolution of each joint angle was $10^{{}^{\circ}}$, then the bitmap of the $C_{space}$ was an array of shape 19×10×19×19×19, i.e., a total of 1,049,760 valid configurations of the manipulator (considering $C_{free}$ and $C_{obs}$). This manipulator was visualized in MapleSoft (Figure 14a); for this visualization and implementation, each link was considered a solid line using 20 midpoints. The workspace contained 20 spherical obstacles of different sizes and two torus obstacles, and we assumed that a guard distance was added to the size of each obstacle for a real robot. By using the dimensions of each link, the range of motion of each joint, and the mathematical representation of each obstacle (torus and spheres), the endpoint’s possible configurations were obtained, as shown in Figure 14b.

**Figure 15:**
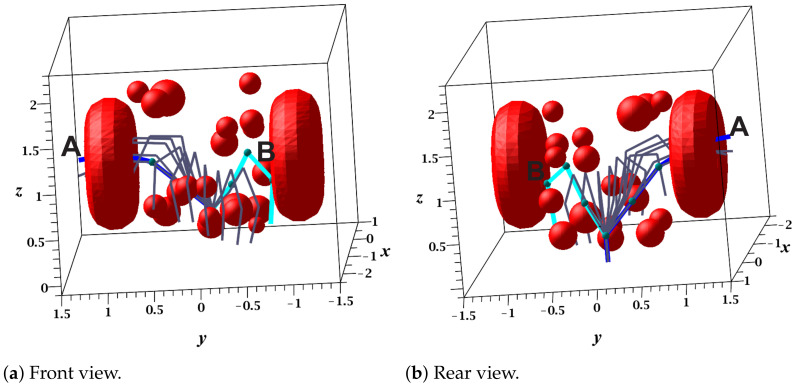
The 5 DOF manipulator’s collision-free path (sequence of configurations). $A=(-50^{{}^{\circ}},-5^{{}^{\circ}},0^{{}^{\circ}},-20^{{}^{\circ}},-20^{{}^{\circ}})$ and $B=(40^{{}^{\circ}},-5^{{}^{\circ}},10^{{}^{\circ}},-80^{{}^{\circ}},-70^{{}^{\circ}})$.

Using a grid size of $10^{{}^{\circ}}$ and the workspace specifications, the RGBPM was able to determine that the $C_{free}$ consisted of 313,958 nodes and 17,127,851 resistors, which modeled the resistive grid. Table 6 shows the main results obtained in the first execution of the RGBPM, where the creation of the bitmap and MNA formulation required 3053.4698 and 37,287.1868 s, respectively. Notably, the time increased considerably compared with the previous three-link case study. However, the number of nodes in $C_{free}$ and the number of neighbors of each node also increased exponentially. Furthermore, the time per iteration in the bitmap creation and MNA formulation processes only increased approximately one order of magnitude compared with the previous study case. This occurred due to the increase in midpoints and obstacles for the bitmap process as well as the increase in neighboring nodes in the MNA formulation, which grew at a rate of ${\left(3\right)}^{n}$, where *n* is the number of DOFs. Figure 15a,b show the front and rear view, respectively, of the solution path for query 1. In this case study, the drop tolerances used for the ILU factorization were α = $1\times 10^{-3}$ and β = $1\times 10^{-6}$.

**Figure 16:**
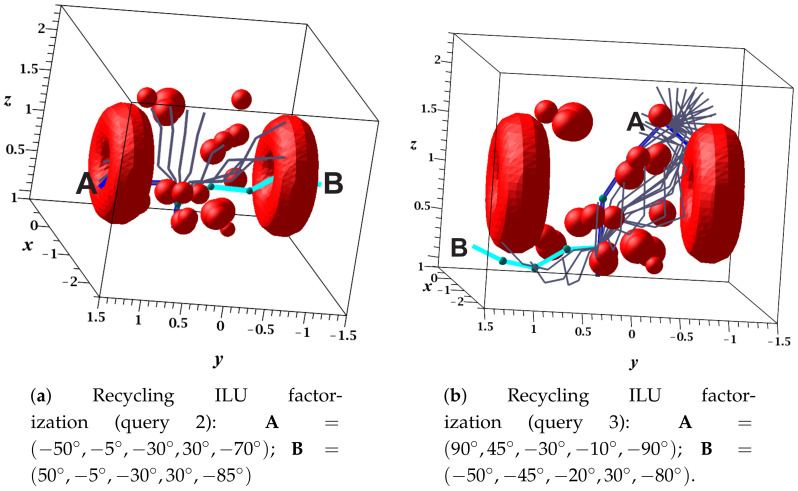
Recycled collision-free paths of the five-DOF manipulator.

Figure 16a,b present the sequence of configurations (successful solution path) of queries 2 and 3, respectively. The results of these queries are shown in Table 7, which show that: (1) Query 2 did not obtain a solution path using the drop tolerance α; however, with β, it did. (2) The drop tolerance value did not determine the length of the path as mentioned in the previous case study. (3) The difference between the execution time of ILU factorization for both drop tolerances were approximately four times, with α being the smallest. (4) Solving the linear system using α was two times faster than using β. (5) The length of the path was directly proportional to the time invested in the execution of the LCC algorithm since the larger number of nodes in the path necessitated a larger number of local comparisons.

### 5.3. Case 3: KUKA Six-DOF Manipulator (Visualization on Maple Soft and V-REP)

**Figure 17:**
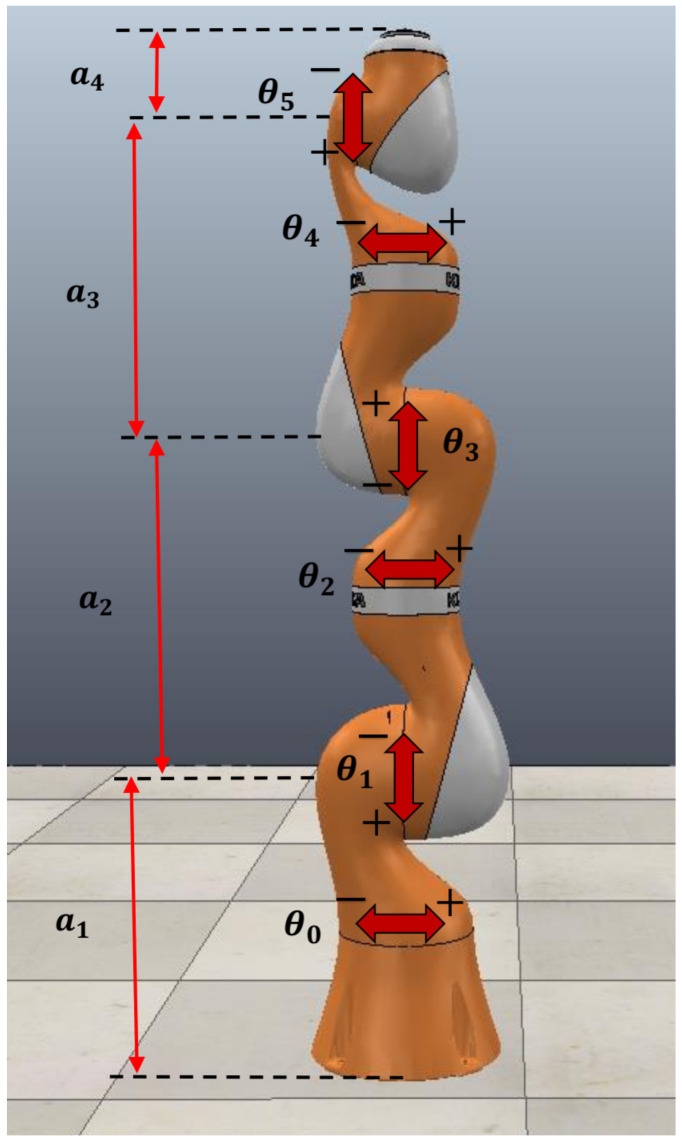
Parameters of the KUKA LBR iiwa 14R820 robot manipulator.

For this case study, the real restrictions and shape of a real industrial manipulator were considered. The selected robot was the KUKA LBR iiwa 14R820, which is a seven-DOF manipulator, where the last DOF is a rotation joint for the end-effector. To only plan a path for the manipulator’s links, the end-effector is ignored, so the robot is considered only as a 6 DOF manipulator. Figure 17 presents the configuration and joint position of the manipulator. The Denavit–Hartenberg parameters, dimensions of each link, and the full motion range of each angle joint are listed in Table 8. For this case study, only a motion range of $\pm 90^{{}^{\circ}}$ was used for each angle and the resolution for $\theta_{0},\theta_{1},\theta_{2},$ and $\theta_{4}$ was $15^{{}^{\circ}}$; for $\theta_{3}$, $30^{{}^{\circ}}$; and for $\theta_{5}$, $60^{{}^{\circ}}$. This grid resolution was selected to restrict the physical memory needed to store the MNA matrix and the incomplete LU factors. Then, the bitmap of the $C_{space}$ was an array of shape 12×12×12×6×12, i.e., a total of 373,248 valid configurations of the manipulator (considering $C_{free}$ and $C_{obs}$).

**Figure 18:**
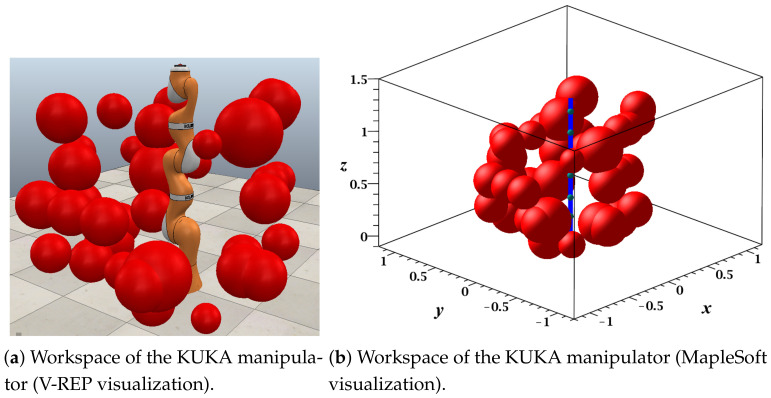
Workspace and configuration space of the KUKA 6 DOF manipulator: Maple Soft and V-REP visualization.

**Figure 19:**
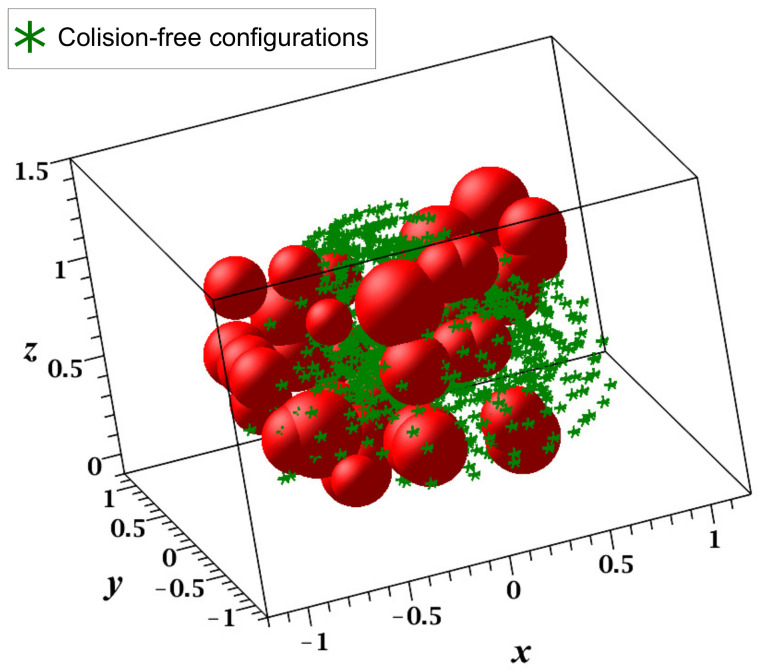
Configuration space of the KUKA manipulator (MapleSoft visualization).

This case study was visualized in MapleSoft and V-REP (Figure 18a,b); in this experiment, each link was considered a solid line using 20 midpoints. The workspace contained a total of 40 different-sized spherical obstacles, and the guard distance was 0.1 m, which was considered in the size of each obstacle. Figure 19 shows all points reachable by the endpoint of the last link of the manipulator.

Using the grid size previously defined and the workspace specifications including the robot dimensions, motion angle range, and obstacle specifications (positions, dimension, and shapes), the RGBPM was able to determine that the $C_{free}$ consisted of a total of 204,087 nodes and 30,335,568 resistors, which modeled the resistive grid. The possible configurations of the endpoint for this manipulator are presented in Figure 19.

**Figure 20:**
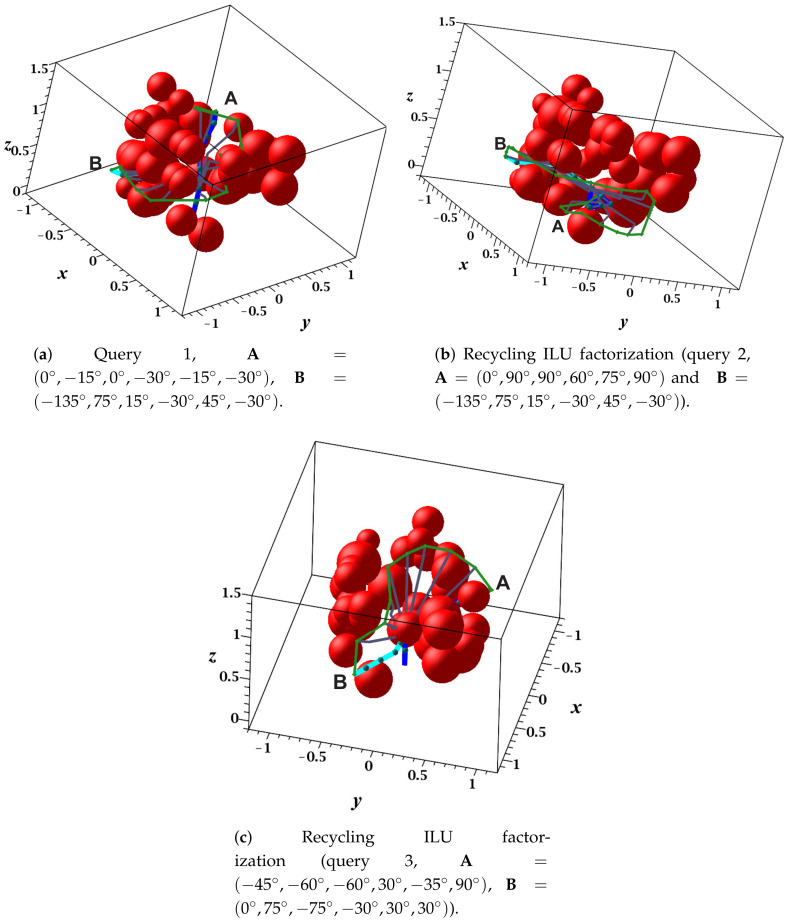
Collision-free path for a KUKA manipulator: MapleSoft visualization.

Table 9 provides the main results obtained in the first execution of the RGBPM. For this case, the creation of the bitmap and the MNA formulation required 2724.4385 and 37,379.0282 s, respectively. In this case study, compared with the previous five-DOF manipulator, the number of nodes in $C_{free}$ is lower; however, due to the number of DOFs of the KUKA robot, three times more neighbors must be manipulated in the bitmap creation process, MNA formulation, and LCC search algorithm. Figure 20a–c show the visualization of the sequence of the solution paths in MapleSoft of queries 1, 2, and 3, respectively. For this case study, the drop tolerances in the ILU factorization were α = $1\times 10^{-6}$ and β = $1\times 10^{-9}$.

The results of queries for the KUKA case study are shown in Table 10. In this table, the following can be identified: (1) Query 1 did not obtain a solution path using the drop tolerance α; however, with β, it did. (2) For query 2, the same path was obtained using α and β tolerances, i.e., the value of the drop tolerance did not determine the length of the path as mentioned in the previous case studies. (3) The difference between the execution time of ILU factorization for both drop tolerances was almost the same, with α being the smallest. Additionally, something similar was observed for the time required to obtain the linear system solution, where only for the third query was the execution time for α three times greater than that of β. (4) The length of the path was directly proportional to the time invested in the execution of the LCC algorithm since the larger number of nodes in the path and the higher number of DOFs necessitated a larger number of local comparisons.

**Figure 21:**
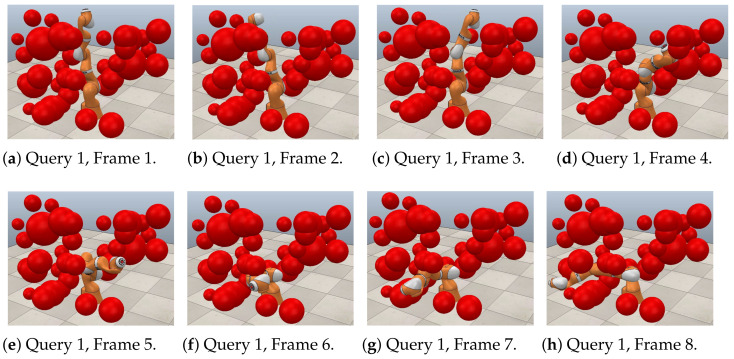
Collision-free path for a KUKA manipulator. Query 1, $A=(0^{{}^{\circ}},-15^{{}^{\circ}},0^{{}^{\circ}},-30^{{}^{\circ}},-15^{{}^{\circ}},-30^{{}^{\circ}})$ and $\;B=(-135^{{}^{\circ}},75^{{}^{\circ}},15^{{}^{\circ}},-30^{{}^{\circ}},45^{{}^{\circ}},-30^{{}^{\circ}})$.

**Figure 22:**
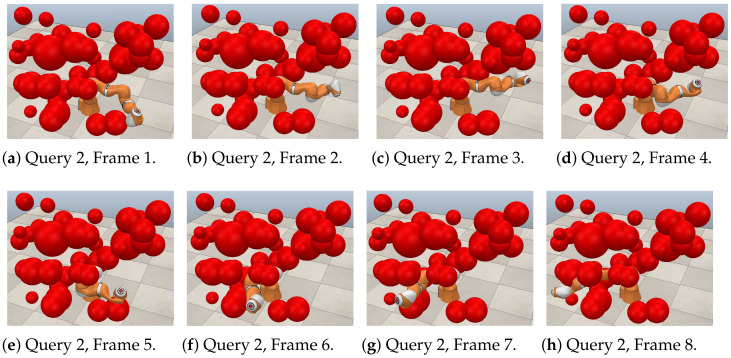
Collision-free path for a KUKA manipulator. Query 2, $A=(0^{{}^{\circ}},90^{{}^{\circ}},90^{{}^{\circ}},60^{{}^{\circ}},75^{{}^{\circ}},90^{{}^{\circ}})$ and $\;B=(-135^{{}^{\circ}},75^{{}^{\circ}},15^{{}^{\circ}},-30^{{}^{\circ}},45^{{}^{\circ}},-30^{{}^{\circ}})$.

**Figure 23:**
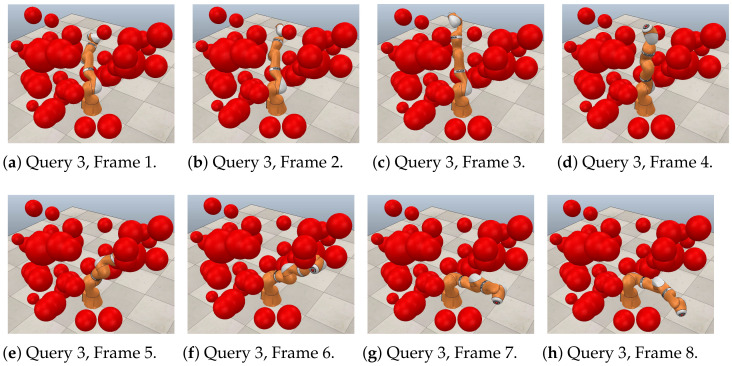
Collision-free path for a KUKA manipulator. Query 3, $A=(-45^{{}^{\circ}},-60^{{}^{\circ}},-60^{{}^{\circ}},30^{{}^{\circ}},-35^{{}^{\circ}},90^{{}^{\circ}})$ and $\;B=(0^{{}^{\circ}},75^{{}^{\circ}},-75^{{}^{\circ}},-30^{{}^{\circ}},30^{{}^{\circ}},30^{{}^{\circ}})$.

Figure 21, Figure 22 and Figure 23 show a sequence of eight frames for each query in this case study. Each was executed in V-REP using the paths obtained through RGBPM. In each of these trajectories, due to the consideration of the guard distance, the manipulator does not collide at any time with the obstacles in the environment. Each of these sequences corresponds to those presented in Figure 20a–c, respectively. In the MapleSoft visualization, the obstacles look bigger because each considers the guard distance in its size since the robot model is a representation using segments of straight lines.

**Figure 24:**
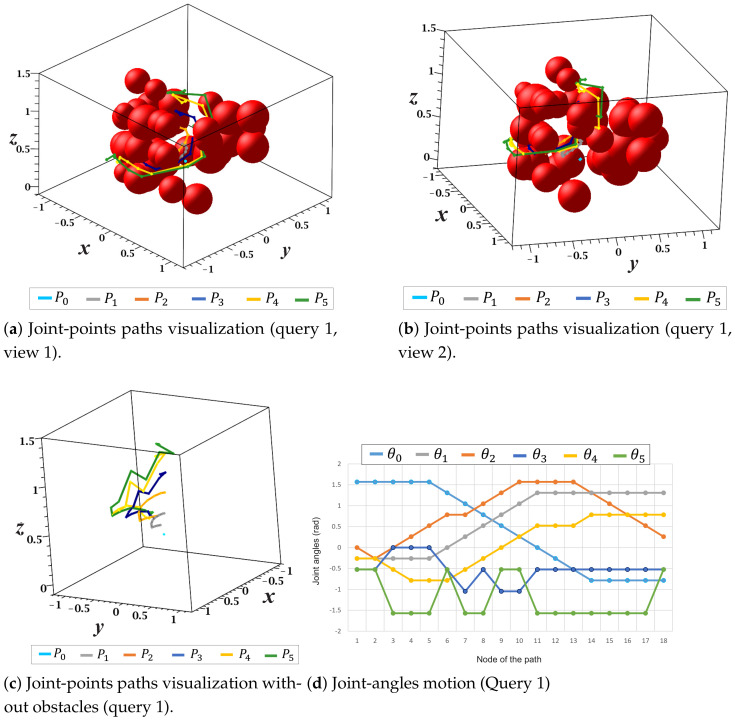
Joint-points paths and joint-angles motion (query 1).

Figure 24 shows a more detailed view of the solution paths of each joint point as well as the path of each motion angle ($\theta_{i}$) of the manipulator for query 1. First, Figure 24a,b show two views of the collision-free paths respective to each joint point $P_{i}$. In these figures, none of the paths collide with obstacles in the environment. Second, Figure 24c presents a complete view of each joint point’s solution paths without the representation of obstacles. Finally, Figure 24d shows the changes in each manipulator motion angle with respect to each node of the solution path.

**Figure 25:**
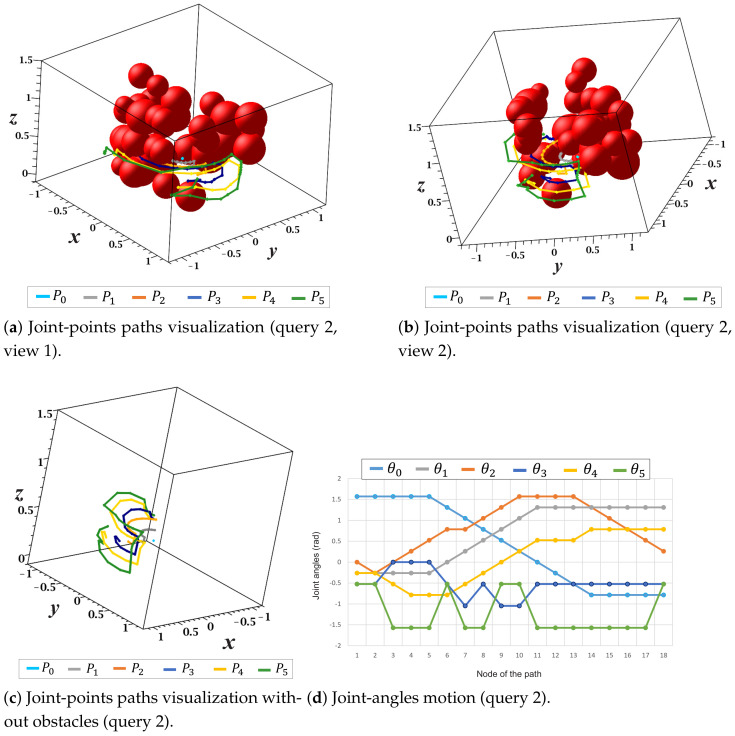
Joint-points paths and joint-angles motion (query 2).

More detailed views of the solution paths of each joint point as well as the change in each motion angle ($\theta_{i}$) of the manipulator for query 2 are presented in Figure 25. Figure 25a,b show two views of the collision-free paths respective to each joint point $P_{i}$. In these figures, none of the paths collide with obstacles in the environment. Figure 25c presents a complete view of each joint point’s solution paths without the representation of obstacles. The changes in each manipulator motion angle with respect to each node of the solution path are depicted in Figure 25d.

**Figure 26:**
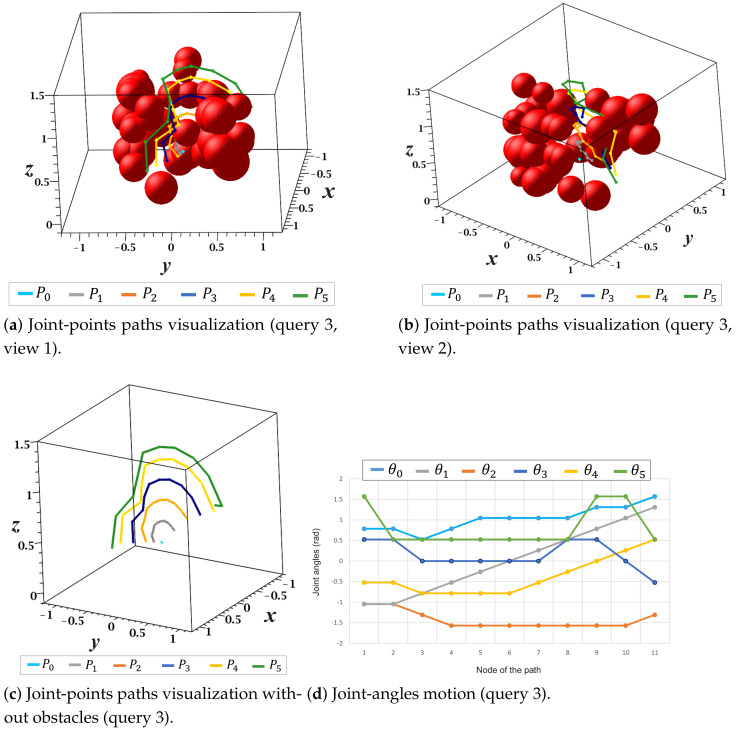
Joint-points paths and joint-angles motion (query 3).

Detailed views of the solution paths of each joint-point and the path of the motion angle ($\theta_{i}$) of each manipulator joint of query 3 are shown in Figure 26. For this case study, Figure 26a,b show two views of the the collision-free paths respective to each joint point $P_{i}$. In these figures, none of the paths collide with obstacles in the environment. Additionally, Figure 26c presents a complete view of each joint-point’s solution paths without the representation of obstacles. The changes in each manipulator’s motion angle with respect to each node of the solution path is depicted in Figure 26d.

From the results of the case studies (Table 3, Table 4, Table 6, Table 7, Table 9 and Table 10), the following was specified: (1) A trade-off exists between configuration space parameters (number of degrees of freedom, number of midpoints, number and shape of obstacles, and grid resolution) and the execution time of critical processes (bitmap creation, MNA formulation, and the LCC algorithm). (2) The number of nodes in the $C_{free}$ determines the size of the MNA matrix, which consequently has a direct impact on the execution time of the ILU factorization and the solution process, even though the number of $C_{free}$ nodes is determined by the number of obstacles. That is, for more obstacles, the MNA will be smaller; however, a smaller number of nodes reduces the chances of finding a solution path. (3) The path’s length is not directly affected by the drop tolerance parameter in the ILU factorization since it is an iterative method; there is no control over the proximity of each value of the solution vector concerning the correct values. However, according to the case study results (Table 4, Table 7, and Table 10), it can be seen that for smaller tolerances, the margin of success of finding a solution path is greater.

## 6. Conclusions and Future Work

In this paper, a novel multiple-query planner inspired by RGBPM was proposed to find collision-free paths for high-DOF manipulators in industrial applications. Through the proposed planner, multiple collision-free paths can be obtained from the data of the first query. In this sense, multiple collision-free paths for different start and goal configurations can be calculated with a reduced computing time (some milliseconds for less than 4 DOF and some seconds for 5 to 6 DOF), with low computational resource use. The effectiveness of the method in solving planning problems in complex environments with multiple static obstacles using high-DOF industrial manipulators was demonstrated in the case studies. Furthermore, a simulation was implemented to validate the proposed multiple-query RGBPM planner’s efficacy in finding feasible paths without collision using a 6 DOF manipulator (KUKA LBR iiwa 14R820) in a complex environment with dozens of surrounding obstacles. These experiments showed that the proposed planner is a powerful tool for industrial applications of high-DOF manipulators in complex 3D environments with multiple static obstacles. Among the main characteristics and advantages of the proposed method over reported sampling-based planners and artificial potential fields planners are: capacity to handle complex environments with dozens of surrounding obstacles, multiple query approach, an extensive strategy to obstacles representations, and easy implementation on industrial manipulators through D-H parametrization.

For future work, some modifications remain to be implemented to improve the performance of the proposed multiple-query RGBPM. First, the execution time of bitmap creation and MNA formulation can be reduced if a parallelization of these is developed. As shown in Algorithms 1 and 2, the parallelism in these processes is possible due to the for-loops nested in each one. This can be corroborated with the results shown in Table 3, Table 6 and Table 9, where each iteration of bitmap creation consumes less than 0.0001, 0.009, and 0.011 s for cases 1, 2, and 3, respectively; MNA formulation requires less than 0.002, 0.006, and 0.006 s for cases 1, 2, and 3, respectively. Second, in a similar approach, the time spent on ILU factorization can be considerably reduced using parallel ILU factorizations performed on GPUs, as presented in [37,38,39,40].

## Figures and Tables

**Table 1:** D-H parameters of a two-DOF manipulator.

Link *i*	Joint Angle $\theta_{i-1}$	Twist Angle $\alpha_{i-1}$	Length of Linkages (m)	Offset of Linkages (m)	Angle Range
1	$\theta_{0}$	$0^{{}^{\circ}}$	0	$a_{1}=0.3$	$-90^{{}^{\circ}}∼90^{{}^{\circ}}$
2	$\theta_{1}$	$90^{{}^{\circ}}$	$a_{2}=0.5$	0	$-45^{{}^{\circ}}∼45^{{}^{\circ}}$

**Table 2:** D-H parameters of a 3 DOF manipulator.

Link *i*	Joint Angle $\theta_{i-1}$	Twist Angle $\alpha_{i-1}$	Length of Linkages (m)	Offset of Linkages (m)	Angle Range
1	$\theta_{0}$	$0^{{}^{\circ}}$	0	$a_{1}=0.3$	$-90^{{}^{\circ}}∼90^{{}^{\circ}}$
2	$\theta_{1}$	$90^{{}^{\circ}}$	$a_{2}=0.5$	0	$-45^{{}^{\circ}}∼45^{{}^{\circ}}$
3	$\theta_{2}$	$0^{{}^{\circ}}$	$a_{3}=0.5$	0	$-90^{{}^{\circ}}∼90^{{}^{\circ}}$

**Table 3:** Execution time of case study 1 (3 DOF manipulator). ETBC: execution time of bitmap creation, ETPIB: execution time per iteration in the bitmap creation process, ET-MNA: execution time of MNA creation, ETPI-MNA: execution time per iteration in the MNA creation process.

No. Query	ETBC (s)	ETPIB (s)	ET-MNA (s)	ETPI-MNA (s)	No. Nodes	No. Resistors
1	11.4428516	0–0.0009982	6.2851495	0–0.0019949	7291	59,953

**Table 4:** Execution time of case study 1 (3 DOF manipulator). ETF: execution time spent in the incomplete LU factorization, ETS: execution time spent to solve linear system using ILU factors, ETLCC: execution time spent in the LCC algorithm, α: ILU tolerance = $1\times 10^{-3}$, β: ILU tolerance = $1\times 10^{-6}$.

N. Query	ETF (s)|ETS (s) @*α*	ETF (s)|ETS (s) @*β*	ETLCC (s) (*α*|*β*)	Success (*α*|*β*)	Length (*α*|*β*)
1	0.0459111|0.0019632	0.0478875|0.0009972	0.1665551|0.1625701	Yes|Yes	52|53
2	− −|0.0009979	− −|0.0010022	− −|0.1695493	No|Yes	− −|55
3	− −|0.0009975	− −|0.0019773	0.1047202|0.1017282	Yes|Yes	31|32

**Table 5:** D-H parameters of the 5 DOF manipulator.

Link *i*	Joint Angle $\theta_{i-1}$	Twist Angle $\alpha_{i-1}$	Length of Linkages (m)	Offset of Linkages (m)	Angle Range
1	$\theta_{0}$	$0^{{}^{\circ}}$	0	$a_{1}=0.3$	$-90^{{}^{\circ}}∼90^{{}^{\circ}}$
2	$\theta_{1}$	$90^{{}^{\circ}}$	$a_{2}=0.5$	0	$-45^{{}^{\circ}}∼45^{{}^{\circ}}$
3	$\theta_{2}$	$0^{{}^{\circ}}$	$a_{3}=0.5$	0	$-90^{{}^{\circ}}∼90^{{}^{\circ}}$
4	$\theta_{3}$	$0^{{}^{\circ}}$	$a_{4}=0.5$	0	$-90^{{}^{\circ}}∼90^{{}^{\circ}}$
5	$\theta_{4}$	$0^{{}^{\circ}}$	$a_{5}=0.5$	0	$-90^{{}^{\circ}}∼90^{{}^{\circ}}$

**Table 6:** Execution time of case study 2 (5 DOF manipulator). ETBC: execution time of bitmap creation, ETPIB: execution time per iteration in the bitmap creation process, ET-MNA: execution time of MNA creation, ETPI-MNA: execution time per iteration in the MNA creation process.

No. Query	ETBC (s)	ETPIB (s)	ET-MNA (s)	ETPI-MNA (s)	No. Nodes	No. Resistors
1	3053.4698287	0–0.0086051	37,287.1868509	0–0.0059847	313,958	17,127,851

**Table 7:** Executiontime for case study 2 (5 DOF manipulator). ETF: execution time spent for the incomplete LU factorization, ETS: execution time spent to solve the linear system using ILU factors, ETLCC: execution time spent in the LCC algorithm, α: ILU tolerance = $1\times 10^{-3}$, β: ILU tolerance = $1\times 10^{-6}$.

No. Query	ETF (s)|ETS (s) @*α*	ETF (s)|ETS (s) @*β*	ETLCC (s) (*α*|*β*)	Success (*α*|*β*)	Length (*α*|*β*)
1	1521.8933052|0.2214086	6044.8255501|0.4538076	14.2039926|14.2133202	Yes|Yes	16|14
2	− −|0.1456099	− −|0.4757362	− −|16.297971	No|Yes	− −|13
3	− −|0.1845103	− −|0.4737734	45.4964143|50.0593325	Yes|Yes	34|36

**Table 8:** D-H parameters of the KUKA 6 DOF manipulator.

Link *i*	Joint Angle $\theta_{i-1}$	$\alpha_{i-1}$; T, A.	Length of Linkages (m)	Offset of Linkages (m)	Angle Range
1	$\theta_{0}$	$90^{{}^{\circ}}$	0	$a_{1}=0.36$	$-170^{{}^{\circ}}∼170^{{}^{\circ}}$
2	$\theta_{1}$	$-90^{{}^{\circ}}$	$a_{2}/2=0.21$	0	$-120^{{}^{\circ}}∼120^{{}^{\circ}}$
3	$\theta_{2}$	$-90^{{}^{\circ}}$	$a_{2}/2=0.21$	0	$-170^{{}^{\circ}}∼170^{{}^{\circ}}$
4	$\theta_{3}$	$90^{{}^{\circ}}$	$a_{3}/2=0.2$	0	$-120^{{}^{\circ}}∼120^{{}^{\circ}}$
5	$\theta_{4}$	$90^{{}^{\circ}}$	$a_{3}/2=0.2$	0	$-170^{{}^{\circ}}∼170^{{}^{\circ}}$
6	$\theta_{5}$	$-90^{{}^{\circ}}$	$a_{4}=126$	0	$-120^{{}^{\circ}}∼120^{{}^{\circ}}$

**Table 9:** Execution time of case study 3 (KUKA six-DOF manipulator). ETBC: execution time of bitmap creation, ETPIB: execution time per iteration in the bitmap creation process, ET-MNA: execution time of MNA creation, ETPI-MNA: execution time per iteration in the MNA creation process.

No. Query	ETBC (s)	ETPIB (s)	ET-MNA (s)	ETPI-MNA (s)	No. Nodes	No. Resistors
1	2724.4385299	0–0.0100044	37,379.0282342	0–0.0059834	204,087	30,335,568

**Table 10:** Execution time of case study 3 (KUKA 6 DOF manipulator). ETF: execution time spent for the incomplete LU factorization, ETS: execution time spent to solve linear system using ILU factors, ETLCC: execution time spent in the LCC algorithm, α: ILU tolerance = $1\times 10^{-6}$, and β: ILU tolerance = $1\times 10^{-9}$.

N. Query	ETF (s)|ETS (s) @*α*	ETF (s)|ETS (s) @*β*	ETLCC (s) (*α*|*β*)	Success (*α*|*β*)	Length (*α*|*β*)
1	7185.3962328|0.8457383	8421.5324378|0.9041367	− −|24.8436352	No|Yes	− −|18
2	− −|0.8387568	− −|0.8330152	35.4426557|43.276417	Yes|Yes	28|28
3	− −|2.1003303	− −|0.8520035	17.8586804|23.271181	Yes|Yes	11|12

## Data Availability

Not applicable.
